# Supplementary figures and images for: Adolescent Normative Intervals for Body Surface Gastric Mapping: Spectral Analysis
Source: Neurogastroenterol Motil. 2026 Mar 11;38(3):e70282. doi: 10.1111/nmo.70282 (PMC12979704; doi:10.1111/nmo.70282)

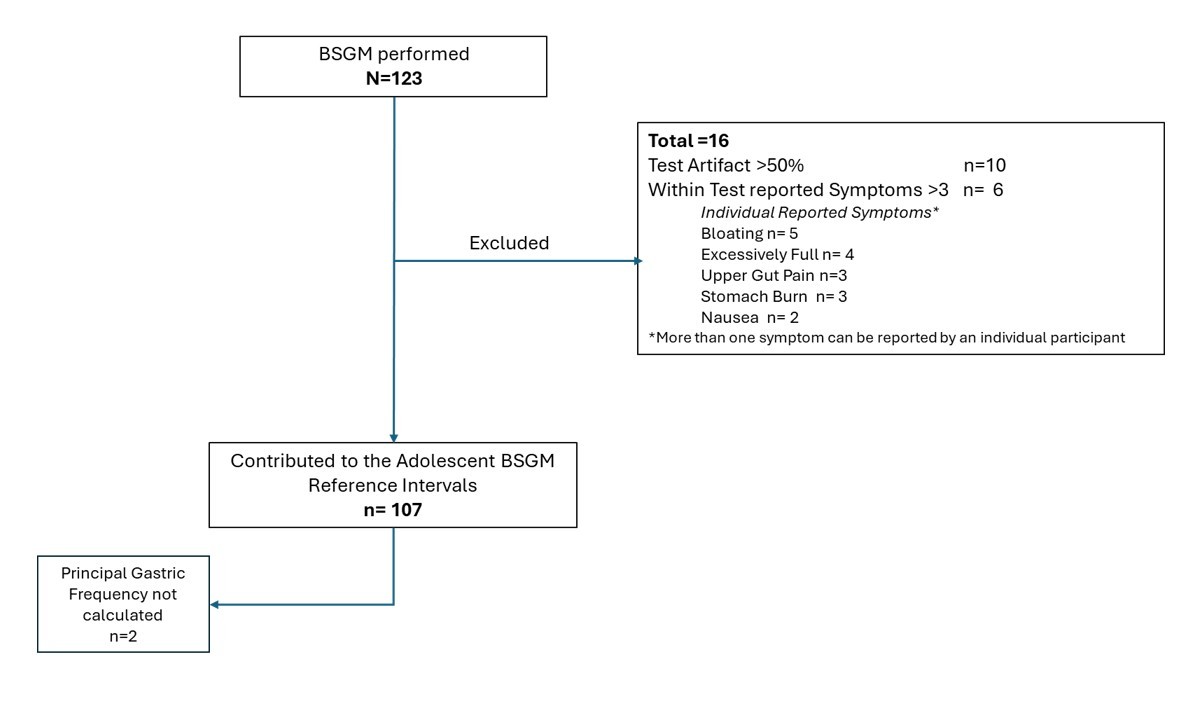

Supplement: Supplementary file 1 — Figure S1: nmo70282‐sup‐0001‐FigureS1.jpg. [file NMO-38-e70282-s001.jpg]

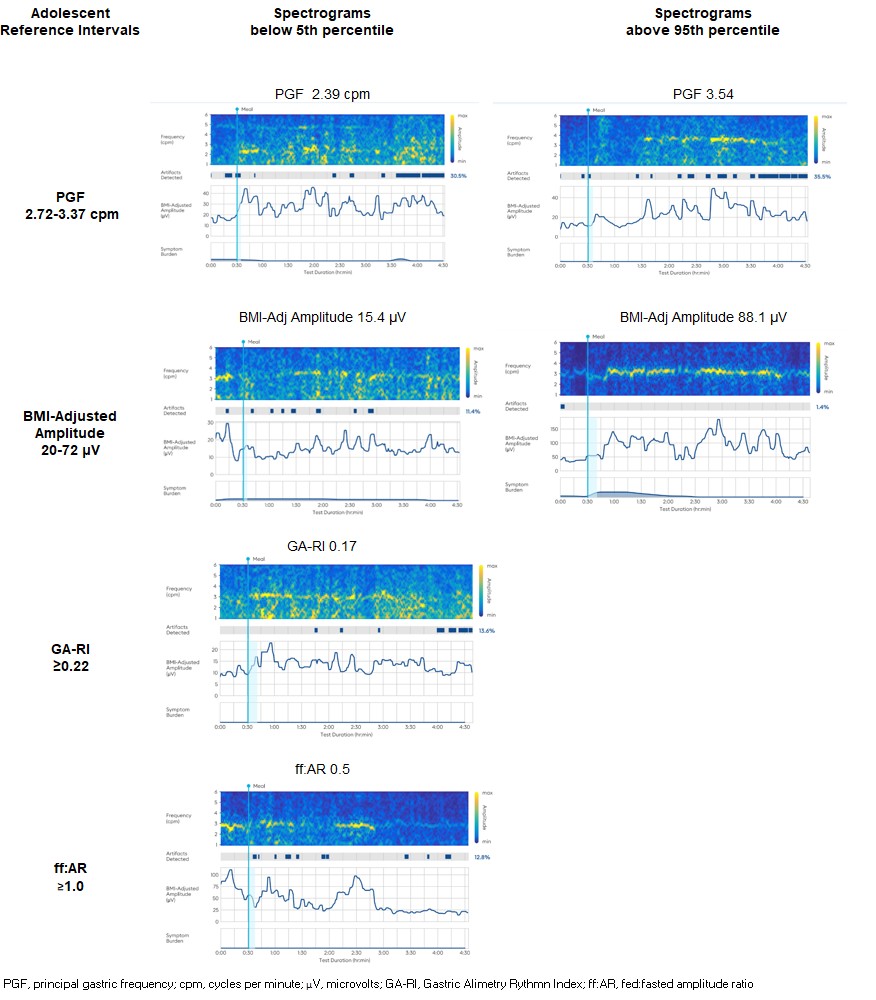

Supplement: Supplementary file 2 — Figure S2: nmo70282‐sup‐0002‐FigureS2.jpg. [file NMO-38-e70282-s005.jpg]
